# Field efficacy of pyrethroid treated plastic sheeting (durable lining) in combination with long lasting insecticidal nets against malaria vectors

**DOI:** 10.1186/1756-3305-3-65

**Published:** 2010-08-03

**Authors:** Fabrice Chandre, Roch K Dabire, Jean-Marc Hougard, Luc S Djogbenou, Seth R Irish, Mark Rowland, Raphael N'Guessan

**Affiliations:** 1 Institut de Recherche pour le Développement (IRD), UR016, Caractérisation et Contrôle des Populations de Vecteurs, UMR224 MIVEGEC, BP 64501, 34394 Montpellier cedex 5, France; 2 Centre de Recherche Entomologique de Cotonou(CREC), 06 BP 2604 Cotonou, République du Bénin; 3 Institut de Recherche en Sciences de la Santé (IRSS)/Centre Muraz (CM), 01 BP 390, Bobo-Dioulasso, Burkina Faso; 4 Institut de Recherche pour le Développement (IRD), BP 1386 Dakar, République du Sénégal; 5 Institut Régional de Santé Publique, Ouidah, 918 Cotonou, République du Bénin; 6 Department of Infectious & Tropical Diseases, London School of Hygiene and Tropical Medicine, Keppel Street, London, WC1E 7HT, UK

## Abstract

**Background:**

Insecticide treated plastic sheeting (ITPS), sometimes known as *durable lining*, has potential as a long-lasting insecticidal surface for malaria vector control when used as lining for interior walls and ceilings inside the home. Against a backdrop of increasing long lasting net (LN) coverage, we examined the effect of combining permethrin-treated plastic sheeting (ITPS) with LNs in Burkina Faso.

**Methods:**

A verandah trap experimental hut trial of ITPS with or without Olyset LN was conducted in the Vallée du Kou near Bobo-Dioulasso, where the two molecular forms of *Anopheles gambiae s.s*., S (frequency 65%) and M (frequency 35%), occur. The S form is mostly pyrethroid resistant (F_kdr _= 92%) owing to the *kdr *mechanism, and the M form is mostly *kdr *susceptible (F_kdr _= 7%). The treatment arms included ITPS, Olyset, ITPS plus Olyset, ITPS plus untreated net (with or without holes), and untreated control.

**Results:**

ITPS was significantly inferior to Olyset LN in terms of mortality (37% vs 63%), blood feeding inhibition (20% vs 81%) and deterrence (0 vs 42%) effects, and hence altogether inferior as a means of personal protection (16% vs 89%). The addition of ITPS to Olyset did not improve mortality (62%), blood feeding inhibition (75%), deterrence (50%) or personal protection (88%) over that of Olyset used alone. Use of untreated nets - both holed and intact - with ITPS provided greater protection from blood-feeding. The intact net/ITPS combination killed more mosquitoes than ITPS on its own.

**Conclusions:**

Although ITPS has a potential role for community control of malaria, at low coverage it is unlikely to be as good as Olyset LNs for household protection. The combination of pyrethroid IRS and pyrethroid LN - as practiced in some countries - is unlikely to be additive except, perhaps, at high levels of IRS coverage. A combination of LN and ITPS treated with an alternative insecticide is likely to be more effective, particularly in areas of pyrethroid resistance.

## Background

Insecticide treated plastic sheeting (ITPS) made of laminated polyethylene impregnated with pyrethroid is a promising new tool for malaria prevention and vector control in the domestic environment. Used as a wall covering, ITPS or *durable lining *as it has come to be known may also provide a better aesthetic than mud or cement plaster, the conventional surfaces for insecticide treatment [1]. Laminated polyethylene tarpaulins are also widely used outdoors in emergencies or disasters as temporary shelter for displaced populations, and insecticide treatment seems likely to control malaria vector mosquitoes [2]. Durable lining inside the home can be likened to a long-lasting indoor residual spray treatment (IRS) in which the substrate needs only to be treated once instead of annually. ITPS has been the subject of previous vector control evaluations: outdoors as refugee shelter [2] and indoors as durable lining [3,4]. In the previous evaluation of ITPS in experimental huts in Burkina Faso it showed good effect on the mortality rates of a mixed population of *kdr *resistant and susceptible *Anopheles gambiae *(causing 46% mortality at full coverage) but limited effect on mosquito biting (27% blood feeding inhibition) presumably because the majority of mosquitoes fed on the human occupants before resting on the wall surfaces as is the norm for IRS treatments [3,5]; in IRS campaigns, the reduction of mosquito biting normally comes as a consequence of the reduction in mosquito longevity and population density at higher coverage levels.

Many countries in malaria endemic Africa are currently scaling up the coverage of long lasting insecticidal nets (LNs). This malaria control effort is being supplemented by IRS campaigns in the more endemic foci in some countries. These programmes, funded by the Global Fund or President's Malaria Initiative (PMI) intend to have a lasting impact on malaria transmission [6]. While the impact of high LN coverage on malaria burden is now becoming evident in several African countries where coverage is increasing [7-10], the cost effectiveness of LNs plus IRS combined is presently unknown. In most PMI countries the same category of insecticide as used on LNs (the pyrethroids) is also being used for IRS treatments. It is unclear whether this constitutes a wasteful duplication of resources or a combination intervention that is having double the impact.

The objective of the present study was to further develop the concept of combination interventions by undertaking a new experimental hut trial this time of ITPS and long lasting insecticidal net to examine whether the two interventions together provide improved personal protection and vector control potential compared to ITPS or LN alone.

## Methods

### Study area

The study was carried out in the same location as the study of Diabate *et al. *[3]. The six experimental huts are located in the Vallée du Kou, near Bobo Dioulasso, Burkina Faso. The Vallée du Kou is an area of rice cultivation surrounded by wooded savannah, with a mean annual rainfall of over 1,000 mm. Though few insecticides are used on the rice crop, insecticides are intensively used on nearby cotton fields. There is a rainy season from May to October and a dry season from November to April.

High mosquito biting densities (200 bites/person/night) are recorded during the rainy season. Both M and S molecular forms of *Anopheles gambiae *are found in Vallée du Kou. The M form is predominant but the S form increases in frequency towards the end of the rainy season. The *kdr *mutation occurs in both M and S forms but at different frequencies. These range from 80-100% in the S form and 0-10% in the M form. Low densities of *An. funestus *and *Culex quinquefasciatus *are found inside huts (3-5%)

### Experimental huts

The experimental huts, described by Darriet *et al. *[11], provide an environment for realistic evaluation of the effect of vector control interventions on mortality, feeding rates and behaviour of naturally entering mosquitoes. Each hut measures 2.5 m long, 1.75 m wide, 2 m high, and is positioned 5 m way from adjacent huts. The walls are made of cement and the roofs of corrugated aluminium. A plastic cover is stretched under the roofing as a ceiling to facilitate the catching of mosquitoes. Each hut is surrounded by a water-filled moat to exclude ants and spiders. Entry of mosquitoes is enabled through four, 1 cm wide baffles located on three sides of the hut. The baffles allow entry of mosquitoes but inhibit exiting. A large verandah trap located on the fourth side allows collection of mosquitoes as they attempt to leave the hut.

Six adult male volunteers from the local area slept in the huts each night from 20:00 to 6:00 hours. The sleepers were informed by the local supervisor about the study, and were recruited after giving informed consent. Sleepers were rotated between huts during successive weeks to adjust for any variation in individual attractiveness. In the mornings, each volunteer lowered a curtain between the room and the verandah trap. All mosquitoes in the hut were collected and scored as alive or dead, blood-fed or unfed. Surviving mosquitoes were given access to 10% honey solution and held for a further 24 h to monitor delayed mortality. Any human volunteer showing illness was provided with free medical care and if diagnosed with malaria was treated with artemether/lumefantrine according to WHO guidelines. The present study obtained approval from the ethics committee of Centre Muraz.

Preliminary hut catches were carried out over 18 nights prior to the main study to ensure there were no significant differences in attractiveness between huts.

### Treatments

The six treatments tested in the huts were: 1) untreated control, 2) ITPS alone, 3) holed Olyset net alone, 4) ITPS + holed Olyset net, 5) ITPS + intact untreated net, 6) ITPS + torn untreated net.

Olyset net is a polyethylene net impregnated with the pyrethroid permethrin during manufacture. The netting contains 2% permethrin w/w. The ITPS was a polyethylene sheet also impregnated with permethrin at 2% during manufacture. As the ITPS could not be taken down easily once fixed in place, each treatment was randomly allocated to one of the six huts for the entire duration of the trial.

The treatments were evaluated over 80 nights between mid September and late December 2005 according to the following criteria:

• Deterrence: percentage reduction in the number of mosquitoes caught in a treated hut compared to the number caught in the control hut.

• Induced exiting: percentage of mosquitoes caught in the verandah trap relative to the control.

• Blood-feeding rate: percentage of all mosquitoes in the hut that were blood fed.

• Overall mortality: percentage of mosquitoes in the hut dead after 24 h.

As the personal protection provided by a treatment stems not only from reduction in blood-feeding rate but also from deterrence, the personal protection was calculated using the formula:

Where B_0 _is total bloodfeeding in the control hut and B_t _is the total bloodfeeding in the treatment hut.

The overall insecticidal effect takes into consideration the number killed relative to the total entering the control hut correcting for death by natural causes, estimated by:

Where K_t _is the total number dead in the treatment hut, K_u _is the number dead in the control hut, and T_u _is the total number collected in the control hut.

### Genetic information

Genomic DNA was extracted from field collected mosquitoes and PCR amplified to determine the molecular forms M or S using the method of Favia *et al. *[12]. Samples of live and dead mosquitoes were taken from all huts except the ITPS + torn untreated net for detection of kdr alleles using the method of Martinez-Torres *et al. *[13].

### Analysis

The hut to hut attractiveness was tested between the six huts using the Kruskal-Wallis test. Because of the non normality in the number of mosquitoes collected from each hut, these data were analysed for each pair of huts using the Mann-Whitney U test. The proportional data (proportions exiting, dying, blood feeding) were analysed using logistic regression (XLSTAT 2006 software). Genotype frequencies were compared using chi-square.

## Results

### Mosquitoes entering the experimental huts

Prior to ITPS installation, 1030 An. gambiae females were collected from the 6 huts over 18 nights. There were no significant differences between huts in the number of mosquitoes collected (P = 0.31), and hence no evidence of differential attractiveness between huts.

During the intervention trial, after installation of ITPS, a total of 422 females of *An. gambiae *were collected in the control hut, an average of 5.3 females per night (Table 1). The majority of females (94%) were blood fed, corresponding to 5.0 bites per person per night. The natural exiting rate was 31.5%, similar to previous trials in Vallée du Kou. Natural mortality was 5.2%.

**Table 1:** Summary of results obtained for *An. gambiae *(n = 80 nights) in the experimental huts.

Hut Treatment	Control	Holed Olyset	ITPS	ITPS + holed Olyset	ITPS + intact untreated net	ITPS + holed untreated net
**Total females caught**	**422**^**ab**^	**246**^**a**^	**443**^**b**^	**211**^**a**^	**315**^**ab**^	**309**^**ab**^
females caught/night	5.3	3.1	5.5	2.6	3.9	3.9
Deterrence (%)	-	41.7	-5.0	50.0	25.4	26.8

**Total females in verandah trap**	**133**	**189**	**352**	**167**	**248**	**221**
Exophily (%)	31.5^**a**^	76.8^**bc**^	79.5^**c**^	79.1^**bc**^	78.7^**bc**^	71.5^**b**^
95% Confidence limits	27.3-36.1	71.1-81.7	75.4-83	73.1-84.1	73.9-82.9	66.2-76.3

**Total females blood fed**	**398**	**43**	**335**	**49**	**89**	**127**
Blood fed (%)	94.3^**a**^	17.5^**b**^	75.6^**c**^	23.2^**bd**^	28.3^**d**^	41.1^**e**^
95% Confidence limits	91.7-96.2	13.2-22.7	71.4-79.4	18-29.4	23.6-33.5	35.7-46.7
Blood feeding inhibition (%)	-	81.5	19.8	75.4	70.0	56.4
Personal protection (%)	-	89.2	15.8	87.7	77.6	68.1

**Total females dead**	**22**	**156**	**164**	**131**	**170**	**103**
Overall mortality (%)	5.2^**a**^	63.4^**b**^	37.0^**c**^	62.1^**bd**^	54.0^**d**^	33.3^**c**^
95% Confidence limits	3.5-7.8	57.2-69.2	32.6-41.6	55.4-68.4	48.4-59.4	27.1-37.4
Corrected for control (%)	-	61.4	33.6	60.0	51.4	29.7
Overall insecticidal effect (%)	-	33.5	35.5	27.3	37.0	20.3

Numbers in the same row sharing a letter superscript do not differ significantly (*P *≥ 0.05)

The reductions in entry rate of 42% and 50% in the huts that contained, respectively, Olyset only and Olyset plus ITPS were not significant (P ≥ 0.05). The entry rates into the huts with ITPS alone or ITPS in combination with untreated nets were also not significantly different from the control.

### Induced exiting

A significant increase in exiting was observed with all treatments ranging from 72% to 80%. These increases corresponded to 2.2 to 2.5 times more females in verandah traps relative to the control.

### Blood feeding inhibition

A significant decrease in blood feeding rates was observed with all treatment arms compared to the control. The blood feeding inhibition rates were highest (75-82%) for the two treatment arms that included Olyset nets but the addition of ITPS to Olyset made no significant difference to the level of inhibition over Olyset alone. Blood feeding inhibition was also high (70%) for the intact untreated net/ITPS combination. The combination of ITPS plus holed untreated net led to a reduced blood feeding inhibition rate (56%) and this fell even further to 20% for ITPS alone.

### Mortality

Mortality rates were significantly higher than the control for all treatment arms. They were highest (62-63%) for the two treatment arms that incorporated Olyset nets, but the addition of ITPS to Olyset made no difference to overall mortality. The mortality decreased to 54% for the ITPS plus intact untreated net combination and decreased still further to 32-37% for ITPS alone or ITPS plus holed untreated net combination.

### In situ bioassays

WHO tests cones were carried out at the beginning and end of the evaluation using mosquitoes of the susceptible reference strain Kisumu. Mortality was systematically 100% for both Olyset and ITPS and was 0% in the control.

### Impact of kdr mutation on mortality

A total of 202 specimens, randomly selected from experimental huts, were genotyped for kdr and molecular forms of *A. gambiae *s.s (Table 2). This population was composed of both M (64.9%) and S (35.1%) forms. The S molecular form recorded a high frequency of the *kdr *allele (F_kdr _= 0.92), the M form recorded a low frequency (F_kdr _= 0.07). The overall frequency of *kdr *was 0.37.

**Table 2:** Mortality of *kdr *genotypes among samples of *A. gambiae *M and S forms collected from the huts (number dead/total collected).

	M form			S form		
	*kdr kdr*	*kdr +*	+ +	*kdr kdr*	*kdr +*	+ +
Control	-	0/1	1/12	0/12	0/1	-
Olyset	-	0/4	18/19	11/20	-	-
ITPS	-	2/2	26/37	2/12	-	0/1
ITPS + untreated net	0/1	3/4	22/24	3/12	-	3/3
ITPS + Olyset	2/2	2/2	21/23	6/9	-	0/1

Among the M form there were insufficient heterozygotes or homozygotes for *kdr *available to detect any trend of differential selection between the ITPS and Olyset LN and most homozygotes for susceptibility were recorded as dead with these treatments. Among the S form few heterozygotes or homozygotes for susceptibility were present, but among the homozygotes for *kdr *significantly more were recorded as dead (55%) with the Olyset treatment than with the ITPS (17%) (P = 0.03). For the ITPS plus untreated net combination the mortality of *kdr/kdr *was 25%, closer to that of ITPS alone, whereas for the ITPS plus Olyset combination, the mortality of *kdr/kdr *was 67%. These trends are consistent with overall mortality of *A. gambiae *recorded with ITPS and Olyset (Table 1) and confirm that Olyset is able to kill many homozygotes for *kdr *in the S molecular form.

## Discussion

The experimental hut trial was designed to test whether the combination of ITPS plus LN was a significantly improvement over LN alone. The rationale was clear: Host-seeking mosquitoes entering a hut may alight on the walls prior to approaching the sleeping host, or may return to the walls if blocked by a mosquito net, or if successful in feeding may rest on the walls while digesting the blood meal. Hence when ITPS and LNs are used together there are multiple opportunities for mosquitoes to be killed by the insecticide either before or after feeding. It was predicted that the combination of ITPS and LN would show the greatest effect on mortality and blood feeding inhibition. Surprisingly the results did not match this prediction.

In the previous study of ITPS, performed at the same experimental station, the effectiveness of ITPS was correlated with the surface area covered [3]. With 4 walls covered, the main effect was on mortality with 45% of females killed, whereas the effect on blood feeding inhibition was limited, recording only 19% inhibition compared to the control. Similar results to these were obtained with ITPS in the present study, confirming that ITPS has the potential to confer community protection if used by the majority but only giving limited personal protection when used by a few. The addition of an untreated mosquito net to ITPS failed to increase deterrence or egress, but both the torn and especially the intact untreated net improved personal protection, as expected. The addition of the untreated intact net significantly increased the mortality due to ITPS. This might be due to dessication of females that were unable to feed or to 'frustrated' females making repeated flights between walls and netted sleeper which increases the chances of picking up a lethal dose of insecticide from ITPS surfaces compared to mosquitoes that were able to feed through the holed nets more readily and then fly to the verandah.

The holed Olyset net had a strong effect on blood feeding inhibition owing to the excito-repellent effect of permethrin which prevented mosquitoes from penetrating the holes or feeding on body parts pressed against the netting. A significant impact on mortality was observed with Olyset, with 61% being killed, many of which were homozygous for *kdr *resistance. Mortality was much greater with Olyset alone than with ITPS alone. Pick up of pyrethroid from the net is presumably enhanced among mosquitoes that persist in host seeking behaviour than among mosquitoes that alight on the ITPS and are then repelled into the verandah.

The most significant finding of this study was the lack of additive effect when ITPS was combined with LN over that of LN alone. It was anticipated that ITPS would kill some of the mosquitoes which were repelled from the net or which had succeeded in blood-feeding through the torn Olyset but then alighted on the walls. Surprisingly, the impact of Olyset plus ITPS over Olyset alone was not significantly different with respect to deterrence, egress, blood-feeding or mortality. The personal protection with a torn Olyset net was just as high without as with ITPS. The overall insecticidal effect was nearly the same between ITPS and Olyset alone and was actually lower when the two were combined. This indicates the ITPS added no benefit to the ITN. This is not to say ITPS had no effect, because its effect could be seen in the hut that had ITPS alone. However, there was no additional effect when the two were used together.

The failure of the combination appears to be due to Olyset masking any effect from ITPS presumably because both materials were treated with the same insecticide. The mosquitoes that survived contact with permethrin on Olyset were either unaffected by permethrin on ITPS or took refuge in the verandah trap.

The other important implication of the trial relates to the renewed interest in applying IRS alongside use of LNs, as supported by the President's Malaria Initiative. ITPS on four walls and ceiling acts much like an IRS treatment. In PMI countries distribution of LNs and IRS are being done together [6]. If LNs are sufficient by themselves or better than IRS at killing genotypes for resistance (as appears to be the case from the genotyping of samples from our trial) there seems little to be gained from spraying pyrethroids on walls too. The only justification for combining the interventions would be if the IRS had greater community impact over LNs alone. An experimental hut trial cannot provide this evidence. The benefit for combining IRS with LNs appears to hold true in some countries, where the combination gave greater protection against malaria than either intervention when used alone [14].

There is debate over whether ITNs or LNs are less likely to select for resistance than IRS. The hut trial indicated that LNs are more likely to kill homozygotes for *kdr *resistance than ITPS are. However both LNs and ITPS caused differentially mortality of susceptible +/+ and *kdr*/+ relative to *kdr/kdr *and hence both methods are likely to select for resistance. A field evaluation conducted in experimental huts from Cotonou, in south Benin, showed that nets treated with permethrin at various concentration (from 50 to 1000 mg/m^2^) did not select for *kdr *mutation [15]. However such selection might be much less evident in Cotonou since the mortality rates were very low compared to the present study. Two hypotheses could explain the differences of mortality between both studies. Firstly, there was a significantly higher excito-repellent effect of permethrin EC formulation used in Cotonou compared with Olyset LN as indicated by the very high deterrence (>83%) and induced exiting (4-12 fold more than in control) in Cotonou study. Such a high excito-repellent effect reduced the selection pressure of permethrin by preventing the contact of mosquitoes with treated nets. Secondly, a high resistance level of *An. gambiae *in Cotonou, associating kdr with metabolic resistance, that reduces the efficacy of pyrethroids treated nets as suggested by the study of N'Guessan *et al. *[16].

Combinations of unrelated insecticides may yield greater benefit in terms of protection or mass killing effect. A combination of pyrethroid treated net and a non-irritant ITPS could prove additive if mosquitoes stayed in prolonged contact with lined walls. Nets treated with pyrethroid plus carbamate or organophosphate combinations show good effect even against pyrethroid resistant *An. gambiae *or *Culex quinquefasciatus *[5,17,18]. Further trials should examine alternative insecticides applied as IRS or ITPS with different modes of action to pyrethroids to see whether an additive effect is possible with LNs [19]. As resistance to pyrethroids has become prevalent throughout West Africa and continues to spread [20-22] the combination of non pyrethroid ITPS and pyrethroid LNs might have a future in sustaining control whilst 'living with pyrethroid resistance'.

The other context in which ITPS has potential is during complex emergencies or natural disasters [23]. A combination of ITPS as temporary shelter together with materials such as blankets or sheets treated with long lasting repellent or insecticide [2,24] might have a positive outcome, especially if the treatments have independent modes of action. After the emergency, during the rehabilitation process, the ITPS might even be used to good protective effect for the lining of interior walls of new homes.

## Competing interests

The authors received financial support from Sumitomo Chemical Co. to conduct the study but have no competing or commercial interests with the manufacturer.

## Authors' contributions

FC, JMH designed and supervised the study, data analysis and drafted the manuscript. RN'G, RKD conducted the field trials and bioassays and drafted the manuscript. LD did the molecular laboratory study and revised the manuscript. MR, SRI participated to the data analysis & interpretation and they drafted the manuscript. All co-authors read and approved the final manuscript.
